# Genetic differentiation and phylogeography of Erythroneurini (Hemiptera, Cicadellidae, Typhlocybinae) in the southwestern karst area of China

**DOI:** 10.1002/ece3.11264

**Published:** 2024-04-10

**Authors:** Guimei Luo, Tianyi Pu, Jinqiu Wang, Weiwei Ran, Yuanqi Zhao, Christopher H. Dietrich, Can Li, Yuehua Song

**Affiliations:** ^1^ School of Karst Science Guizhou Norml University/State Engineering Technology Institute for Karst Desertification Control Guiyang Guizhou China; ^2^ Guizhou Provincial Key Laboratory for Rare Animal and Economic Insect of the Mountainous Region Guiyang University Guiyang Guizhou China; ^3^ Illinois Natural History Survey, Prairie Research Institute University of Illinois Champaign Illinois USA

**Keywords:** ancestral area reconstruction, Erythroneurini, genetic structure, MaxEnt model, mitochondrial genome, phylogeography

## Abstract

Erythroneurini is the largest tribe of the microleafhopper subfamily Typhlocybinae. Most prior research on this tribe has focused on traditional classification, phylogeny, and control of agricultural pests, and the phylogeography of the group remains poorly understood. In this study, the mitochondrial genomes of 10 erythroneurine species were sequenced, and sequences of four genes were obtained for 12 geographical populations of *Seriana bacilla*. The new sequence data were combined with previously available mitochondrial DNA sequence data and analyzed using Bayesian and Maximum‐Likelihood‐based phylogenetic methods to elucidate relationships among genera and species and estimate divergence times. *Seriana* was shown to be derived from within *Empoascanara*. Phylogeographic and population genetic analysis of the endemic Chinese species *Seriana bacilla* suggest that the species diverged about 54.85 Mya (95% HPD: 20.76–66.23 million years) in the Paleogene period and that population divergence occurred within the last 14 million years. Ancestral area reconstruction indicates that *Seriana bacilla* may have originated in the central region of Guizhou, and geographical barriers are the main factors affecting gene flow among populations. Ecological niche modeling using the MaxEnt model suggests that the distribution of the species was more restricted in the past but is likely to expand in the future years 2050 and 2070.

## Graphical abstract

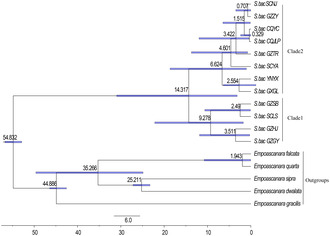

## 1 INTRODUCTION

Leafhoppers (Cicadellidae) are one of the largest families of plant‐feeding insects. They are generally between 3 and 15 mm in length, small in size, widely distributed, and occur in most habitats where vascular plants are found. Many leafhopper species transmit plant‐pathogenic bacteria and viruses that affect various crops, grasslands, forest trees, fruit trees, and other economic plants (Roddee et al., 2018). Adult and juvenile (nymph) leafhoppers usually have similar ecology, living on the aboveground parts of their host plants. Nymphs are capable of jumping but generally do not disperse over long distances. Adults usually have well‐developed wings but relatively few species regularly migrate over long distances. Leafhopper distribution is often constrained by barriers to dispersal such as mountains, water bodies, or regions of unsuitable habitat. Previous studies of leafhopper biogeography (e.g., Cao et al., 2023; Krishnankutty et al., 2016; Wang et al., 2017) have revealed considerable large‐scale biogeographic structure, with many diverse lineages restricted to particular biogeographic regions. Few studies have examined smaller‐scale biogeographic patterns among species within a single genus (e.g., Bernt et al., 2013; Zhang et al., 2016) and studies of phylogeographic patterns within individual leafhopper species have, so far, focused only on agricultural pests (e.g., Akmal et al., 2018; Zhang et al., 2020). Due to their limited dispersal ability, abundance, and specialization on certain host plants and habitats, leafhoppers are ideal subjects for research on speciation and phylogeography (Hill et al., 2009; Marshall et al., 2008). Most research on leafhoppers continues to focus on morphology‐based taxonomy and control of disease vectors and other agricultural pests (Dietrich, 2013). However, additional research is needed to elucidate the particular evolutionary mechanisms that gave rise to the great diversity of leafhopper species. Phylogeography can help improve understanding of the contributions of dispersal and geographic barriers to genetic diversification and speciation in leafhoppers.

Phylogeography combines biogeographic knowledge and molecular biology techniques to reconstruct the historical evolution and formation of different geographic populations among closely related species and within species (Avise et al., 1987; Kumar et al., 2016). Phylogeography has made great contributions to the study of human evolution (Beaumont, 2004), species formation (Kohn, 2005), and biological conservation (O'Brien, 1994). The most widely used molecular markers in phylogeography are in the mitochondrial genome (Clarke et al., 2015; Diedericks & Daniels, 2014; Liu et al., 2016). With the development and improvement of DNA sequencing technology and evolutionary theory, phylogeography has developed rapidly and become an important tool in evolutionary biology (Beheregaray, 2008).

In view of the limited research on the population differentiation and phylogeography of leafhoppers, this study focused on Erythroneurini, which have been well studied from a taxonomic perspective but have not previously included in phylogeographic studies. Based on our extensive field surveys and sampling in the southwest karst regions of China, we conducted phylogenetic analyses to elucidate the relationships among genera and species of this tribe, reconstruct ancestral areas, and estimate divergence times. We then used finer‐scale analyses to further explore the phylogeography of one species apparently endemic to this region. *Seriana bacilla* is a shared species in the southwest and can be used to explore genealogical geography, geological events and climatic fluctuations, population genetic differentiation, historical dynamics, and suitability zones.

## 2 MATERIALS AND METHODS

### 2.1 Specimen collection and DNA extraction

**FIGURE 1:**
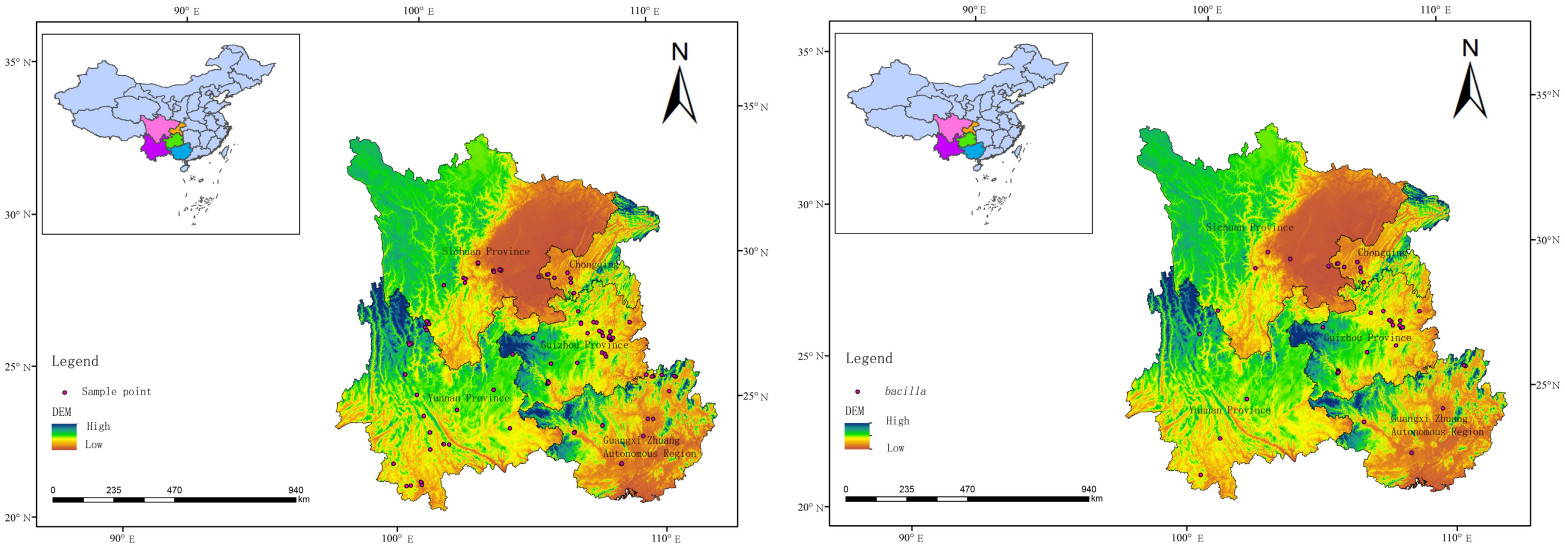
Sample collection and distribution map (the left image shows the distribution of all spotted Erythroneurine specimen collections, and the right image shows the distribution of *Seriana bacilla* specimen collections).

The Specimens used in this study were collected from 25 counties and cities in Guizhou, Yunnan, Sichuan, Guangxi, and Chongqing, situated in the karst environment of southwestern China, by sweeping and light trap (Figure 1). The collected specimens were preserved in 98% ethanol, transported to the laboratory, and stored in a freezer at −20°C. Genomic DNA was extracted from the legs and thorax of each leafhopper specimen using the DNeasy tissue kit following manufacturer's instructions. The extracted DNA was numbered and stored at −20°C after passing the DNA quality test. Whole mitochondrial genome sequences were determined at Berry Genomics (Beijing, China) using an Illumina Novaseq 6000 platform (Illumina, Alameda, CA, USA) by 150 bp paired‐end reads and 5.87 G of raw data were obtained. All specimens are stored in the collection of the School of Karst Science, Guizhou Normal University, China (GZNU).

### 2.2 Mitochondrial genome annotation and analysis

Raw sequence data were assembled using Geneous Prime V 2021.2.2 software and the mitochondrial genome loop splicing was performed manually using Geneous Prime V 2021.2.2 software after determining that the data were correct (Kearse et al., 2012). Genome assembly was performed using GetOrganelle 1.7.5 (parameters: ‐R 10 ‐k 21,45,65,85,105 ‐F animal_mt) (Jin et al., 2020), and the assembly results were blastn (version: BLAST 2.2.30+; parameters: −evalue 1e−5) with the proximal reference mitogenome (accession number NC_047465), and the candidate sequence assembly results were determined based on the comparison. The secondary structure of mitochondrial tRNA genes and 22 tRNA gene positions were determined using the MITOS Web server (Bernt et al., 2013) and the tRNAscan‐SE (Lowe & Eddy, 1997) online website. Contigs were used as input for BLAST searches in GenBank to verify their identity as leafhopper mtDNA sequences (Altschul et al., 1997; Meng et al., 2013; Yu et al., 2017).

### 2.3 Phylogenetic analysis

Two datasets were analyzed. The first, used to estimate relationships among the currently recognized tribes of Typhlocybinae, included sequences of the 13 mitochondrial protein‐coding genes (PCGs). The second included sequences of four mitochondrial genes (COI, COII, Cytb, 16S rRNA) and was used to assess phylogenetic relationships between species of the genera *Seriana* and *Empoascanara*. The latter dataset was also used for population‐level phylogeographic analysis of *S. bacilla*.

The newly obtained gene sequences were aligned and analyzed, and combined with previously available sequence data using PhyloSuite v1.2.2 (Zhang et al., 2020). Sequence alignment was performed using MAFFT v7.4 (Katoh & Standley, 2013). PartitionFinder v2.1.1 (Lanfear et al., 2017) was used to select the best‐fit partitioning scheme for linked nucleotides and the corresponding nucleotide substitution model. Phylogenetic analysis was conducted using the Maximum Likelihood (ML) and Bayesian Inference (BI) methods, as implemented in IQTREE 1.6.5 (Nguyen et al., 2014) and MRBAYES 3.2 (Ronquist et al., 2012), respectively. ML analysis was run using IQ‐TREE for 10,000,000 generations, checking the confidence values of each branch node of the system tree every 1000 times, and BI analysis was run independently using four Markov chain Monte Carlo (MCMC) chains (three heated chains and one cold chain) starting from a random tree; each chain was run for 2 × 10^7^ generations and sampled once every 1000 generations. Tracer v1.7.1 (Rambaut et al., 2018) was used to check for effective sample size (ESS) > 200. Visualization and further processing of phylogenetic trees were performed using FigTree 1.4.3.

### 2.4 Genetic diversity analysis

The genetic diversity of *S. bacilla* was calculated from mitochondrial DNA sequence data by DNAsp5.0 software. In the calculation process, populations were grouped according to collection regions, and since Guangxi and Yunnan were each represented by a single sample, these two collection regions were not counted in the calculation of nucleotide diversity and genetic structure.

### 2.5 Divergence time estimation

Since leafhopper fossils have not yet been included in morphological phylogenetic analysis, we use available fossils to calibrate the root nodes of their respective tribes in order to conservatively estimate the minimum ages of these groups. We used BEAST v2.6.6 (Bouckaert et al., 2014) to estimate the date of origin of Erythroneurini and the divergence time of each species using tandem mitochondrial data under the relaxed log‐normal assumption of the clock and the priori Yule model. Based on the previous molecular timetree of Membracoidea (Christopher et al., 2017) and information on the oldest Membracoidea included in the Paleobiology Database (PDBD) Navigator website (http://paleobiodb.org/navigator/), the maximum age of the root node was constrained with a relaxed lower bound of 174.1 million years ago (Mya) (Yan et al., 2022). Oligo‐Miocene Dominican amber was used to provide minimum age calibration points for Evacanthinae (Dietrich & Vega, 1995). Dominican amber was also used to provide minimum age calibration points for Cicadellinae (Dietrich & Vega, 1995). Therefore, we set four fossil calibration points, (A) root age < 174.1 Mya; (B) 17.5–120 Mya; (C) 17.5–110 Mya; (D) 17.5–90 Mya (normally distributed calibration density; mean = 1 Ma). The Jmodeltest software was used to select the most suitable model, the Substitution Model was selected as the GTR model, the Tree Prior was selected as the Yule process, and the MCMC method was run for 100,000,000 generations, with sampling every 1000 generations (Drummond et al., 2012; Drummond & Rambaut, 2007). Stationarity was assessed using TRACER 1.7.1, with ESS values of >200 taken as evidence of convergence. Maximum clade credibility (MCC) trees were generated using TreeAnnotator v2.4.1 after discarding the first 10% as burn‐in. Figtree software was used to export and visualize the time tree.

### 2.6 Ancestral area reconstruction

To assess the origin and possible dispersal pathways of *S. bacilla* species, a time‐calibrated phylogenetic tree obtained from the BEAST analysis with all outgroups removed and only *S. bacilla* species retained was used as the input tree for ancestral area analysis. BioGeoBEARS (Matzke, 2013) as implemented in RASP v3.2 (Yu et al., 2015) was used to reconstruct ancestral areas and infer their biogeographic history. Specimens representing different *S. bacilla* populations were assigned to eight geographic areas: (A) central Yunnan; (B) northeastern Guangxi; (C) southeastern Sichuan; (D) central Sichuan; (E) western Chongqing; (F) northern Guizhou; (G) northeastern Guizhou; (H) central Guizhou.

### 2.7 Species distribution model

Ecological niche modeling based on the current distribution of *S. bacilla*, we use past, present, and future bioclimatic factors to predict potential distribution areas during the Last Glacial Maximum (LGM, ~22,000 years ago), Mid‐Holocene (~6000 years ago), present and the future years 2050, 2070. *S. bacilla* was first described by our research team in 2020 and its distribution data have mainly been collected from our team through fieldwork over several years. Distribution data of *S. bacilla* were also compiled from relevant theses, dissertations, and other literature, and from China Knowledge Base, 3I Interactive Key and Taxonomic Databases, and Web of Science. ArcGIS 10.7 was used to filter distribution data, and the final dataset contains data from 55 locations. Nineteen bioclimatic variables of paleoclimate are available from the World Climate Dataset v1.4 (http://www.worldclim.org) with resolution of 10 min (Hijmans et al., 2005). Considering the uncertainty of a single climate model, the bioclimatic factors for 2050 and 2070 in the paper use two representative concentration pathways (RCPs) from the CCSM4 model and MIROC‐ESM model, that is, RCP26 and RCP45 (Jiang et al., 2016; Mendlik & Gobiet, 2016; Wang & Chen, 2014). The data obtained were processed by ArcGIS10.7 software, and the MaxEnt model was used to predict suitable areas. Areas are categorized into four different types: non‐suitable area (0–0.07), low suitable area (0.07–0.3), medium suitable area (0.3–0.6), and high suitable area (0.6–1).

To avoid over‐parameterization caused by redundant variables in the model, Pearson correlation analysis was performed on bioclimatic factors using SPSS software. When |*r*| > 0.9, the variable with less biological significance was eliminated. In the end, nine bioclimatic factors were chosen: mean diurnal range (Bio 2), temperature seasonality (Bio 4), min temperature of coldest month (Bio 6), temperature annual range (Bio 7), mean temperature of driest quarter (Bio 9), annual precipitation (Bio 12), precipitation seasonality (Bio 15), precipitation of warmest quarter (Bio 18), precipitation of coldest quarter (Bio 19). Model accuracy was verified using the receiver operating characteristic curve (AUC) of MaxEnt 3.4.1. All models use 25% of distribution information as a test set and 75% as a training set (Phillips & Dudík, 2008).

## 3 RESULTS

### 3.1 Characteristics of the sequences

Data for the 10 newly sequenced species were uploaded to the NCBI database (accession nos. shown in Table 1). Four mitochondrial gene sequences were obtained for *Empoascanara dissimilaris* (COI: OQ469424; COII: OQ506061; Cytb: OQ506062; 16S rRNA: OQ476880). Forty‐eight sequences of four genes (COI, COII, Cytb, 16S rRNA) were obtained for 12 geographical populations of *Seriana bacilla* (Table 2). All sequences have been uploaded to NCBI (COI: OP643508–OP643517, COII: OP675847–OP675856, Cytb: OP649558–OP649567, 16S rRNA: OP646469–OP646478). Among them, from Yuxi of Yunnan Prov. and Tongren of Guizhou Prov., full mitochondrial gene sequences were uploaded with NCBI login numbers OP423027 and OM048922. The characteristics of the sequences are summarized in Table 3.

**TABLE 1:** List of newly sequenced species.

Species	Length (bp)	GenBank No.
*Seriana bacilla*	15,613	OP423027
*Empoascanara quarta*	15,354	OP644293
*Empoascanara defecta*	15,338	OP650002
*Empoascanara plamka*	15,279	OP714122
*Empoascanara alami*	15,255	OP748285
*Empoascanara circumscripta*	15,494	OP747491
*Empoascanara angkhang*	14,928	OP754989
*Empoascanara hongkonica*	15,739	OP754990
*Empoascanara bidenticulata* sp. n.	15,575	OP765234
*Empoascanara falcata*	15,151	OP765235

**TABLE 2:** Information on sample collection from different geographical populations of *Seriana bacilla.*

Region	Code	Location	Sample number	Longitude E	Latitude N	Altitude m	Humidity %	Temperature °C	Acquisition date
Guizhou	S.bac GZZY	Zunyi	27	107°46′38″	27°15′3″	637.33	51	33	2021/8/18
Guizhou	S.bac GZGY	Guiyang	33	106°45′20″	26°19′33″	1080.3	59	29	2021/8/21
Guizhou	S.bac GZSB	Shibing	108	108°4′9″	27°24′	691.1	50	33	2021/8/19
Guizhou	S.bac GZHJ	Huajiang	195	105°39′2″	25°38′40″	1120	70	28	2021/8/25
Guizhou	S.bac GZTR	Tongren	30	108°45′55″	27°49′50″	2572	79	26	2019/7/20
Sichuan	S.bac SCYA	Yaan	12	102°50′13″	29°48′33″	860.05	64	28	2021/8/9
Sichuan	S.bac SCLS	Leshan	52	103°44′18″	29°34′58″	339.38	38	36	2021/8/10
Sichuan	S.bac SCNJ	Neijiang	25	105°15′53″	29°20′34″	376	79	27	2021/8/13
Chongqing	S.bac CQYC	Yongchuan	107	105°53′31″	29°18′8″	417.41	91	23	2021/8/14
Chongqing	S.bac CQJLP	Jiulongpo	16	106°25′11″	29°28′42″	402.73	66	29	2021/8/15
Yunnan	S.bac YNYX	Yuxi	29	102°8′23″	24°40′9″	1630.82	54	28	20,218/1
Guangxi	S.bac GXGL	Guilin	14	110°29′10″	25°46′27″	257	5	32	2021/7/25

**TABLE 3:** Analysis of the composition of the four mitochondrial markers of *Seriana bacilla.*

Feature	COI	COII	Cytb	16S rRNA	mtDNA
Length (bp)	1533	678	1134	1050	4395
A (%)	29.5	36.6	32.2	47	35.5
T (%)	37.7	34.5	37.7	33.8	36.3
G (%)	16.3	14.4	13.6	6.7	13
C (%)	16.5	14.5	16.5	12.5	15.3
AT skew	−0.122	0.030	−0.079	0.163	−0.011
GC skew	−0.006	−0.003	−0.096	−0.302	−0.081
Constant site	1480	643	1087	1027	4237
Variable sites	53	35	47	23	158
Parsimony informative	38	28	30	12	108
Single variant sites	15	7	17	11	50

### 3.2 Phylogenetic analysis

**FIGURE 2:**
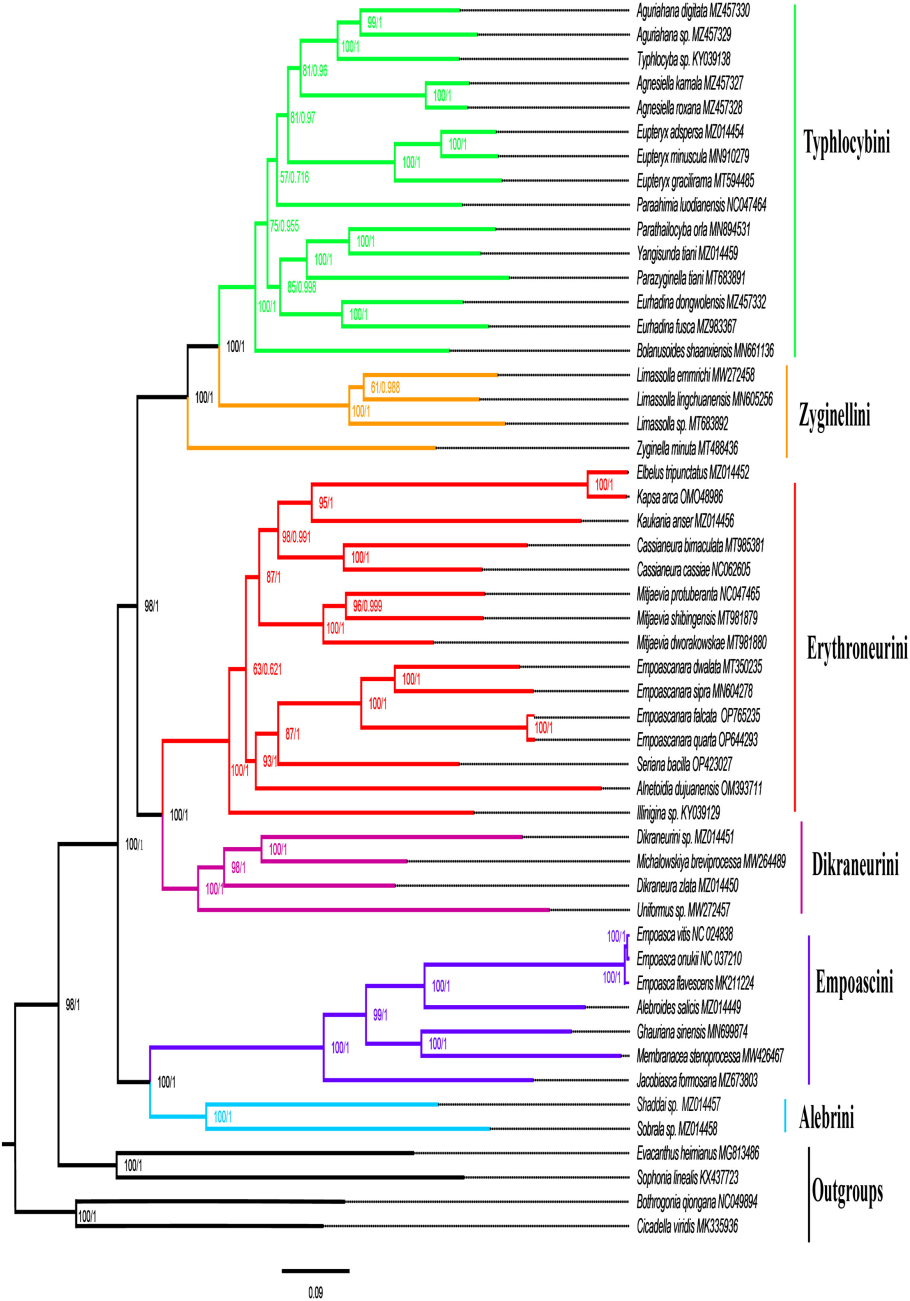
Phylogenetic tree from Typhlocybinae based on nucleotide sequence of 13 PCGs. The first and second numbers to the left of each node denote the ultrafast bootstrap value (UBP) for ML analysis and the Bayesian posterior probability (BPP) for Bayesian inference.

Forty‐seven species representing six tribes of Typhlocybinae were used as the ingroup, and four species representing two other subfamilies were used as outgroups. The species included are listed in Table 4. The ML phylogenetic tree and the BI phylogenetic tree constructed separately based on 13 PCGs showed similar topologies (Figure 2). Phylogenetic trees shows the relationship between these tribes as ((Typhlocybini + Zyginellini) + (Erythroneurini + Dikraneurini)) + (Empoascini + Alebrini). Erythroneurini + Dikraneurini are monophyletic sister groups to each other and Empoascini + Alebrini are also monophyletic. Within Erythroneurini, *Empoascanara* and *Seriana* genera are sister groups. Our results consistently support the monophyly of Alebrini, Dikraneurini, Empoascini, and Erythroneurini.

**TABLE 4:** List of mitochondrial genomes used for phylogenetic analysis of Typhrocybinae in this study.

Tribe	Species	Length (bp)	GenBank No.
Erythroneurini	*Mitjaevia protuberanta*	15,472	NC_047465.1
	*Mitjaevia shibingensis*	15,788	MT981879.1
	*Mitjaevia dworakowskae*	16,399	MT981880.1
	*Empoascanara sipra*	14,827	MN604278.1
	*Empoascanara dwalata*	15,271	MT350235.1
	*Empoascanara quarta*	15,354	OP644293
	*Empoascanara falcata*	15,151	OP765235
	*Seriana bacilla*	15,613	OP423027
	*Illinigina* sp.	14,803	KY039129.1
	*Kapsa arca*	15,594	OMO48986
	*Alnetoidia dujuanensis*	15,375	OM393711
	*Cassianeura cassiae*	15,423	NC_062605.1
	*Cassianeura bimaculata*	14,597	MT985381
	*Kaukania anser*	15,345	MZ014456.1
	*Elbelus tripunctatus*	15,308	MZ014452
Zyginellini	*Paraahimia luodianensis*	16,497	NC_047464.1
	*Parathailocyba orla*	15,382	MN894531.1
	*Limassolla lingchuanensis*	15,716	MN605256.1
	*Limassolla* sp.	17,053	MT683892.1
	*Limassolla emmrichi*	14,677	MW272458.1
	*Zyginella minuta*	15,544	MT488436.1
Typhlocybini	*Eupteryx minuscula*	16,944	MN910279.1
	*Eupteryx adspersa*	15,178	MZ014454
	*Eupteryx gracilirama*	17,173	MT594485
	*Eurhadina dongwolensis*	15,708	MZ457332
	*Eurhadina fusca*	15,302	MZ983367
	*Agnesiella roxana*	15,901	MZ457328
	*Agnesiella kamala*	15,209	MZ457327
	*Aguriahana digitata*	15,854	MZ457330
	*Aguriahana* sp.	15,252	MZ457329
	*Typhlocyba* sp.	15,223	KY039138.1
	*Bolanusoides shaanxiensis*	15,274	MN661136.1
	*Yangisunda tiani*	15,190	MZ014459
	*Parazyginella tiani*	17,562	MT683891
Empoascini	*Empoasca flavescens*	15,152	MK211224.1
	*Empoasca onukii*	15,167	NC_037210.1
	*Empoasca vitis*	15,154	NC_024838.1
	*Ghauriana sinensis*	15,491	MN699874.1
	*Jacobiasca formosana*	16,353	MZ673803
	*Alebroides salicis*	15,890	MZ014449
Alebrini	*Shaddai* sp. SL‐2021a	17,575	MZ014457
	*Sobrala* sp. SL‐2021a	16,732	MZ014458
Dikraneurini	*Michalowskiya breviprocessa*	15,591	MW264489
	*Dikraneura zlata*	15,330	MZ014450
	Dikraneurini sp.	15,306	MZ014451
	*Membranacea stenoprocessa*	14,717	MW426467.1
	*Uniformus* sp.	14,440	MW272457
Evacanthinae	*Sophonia linealis*	13,296	KX437723
	*Evacanthus heimianus*	15,806	MG813486
Cicadellinae	*Bothrogonia qiongana*	15,788	NC_049894
	*Cicadella viridis*	15,891	MK335936

**FIGURE 3:**
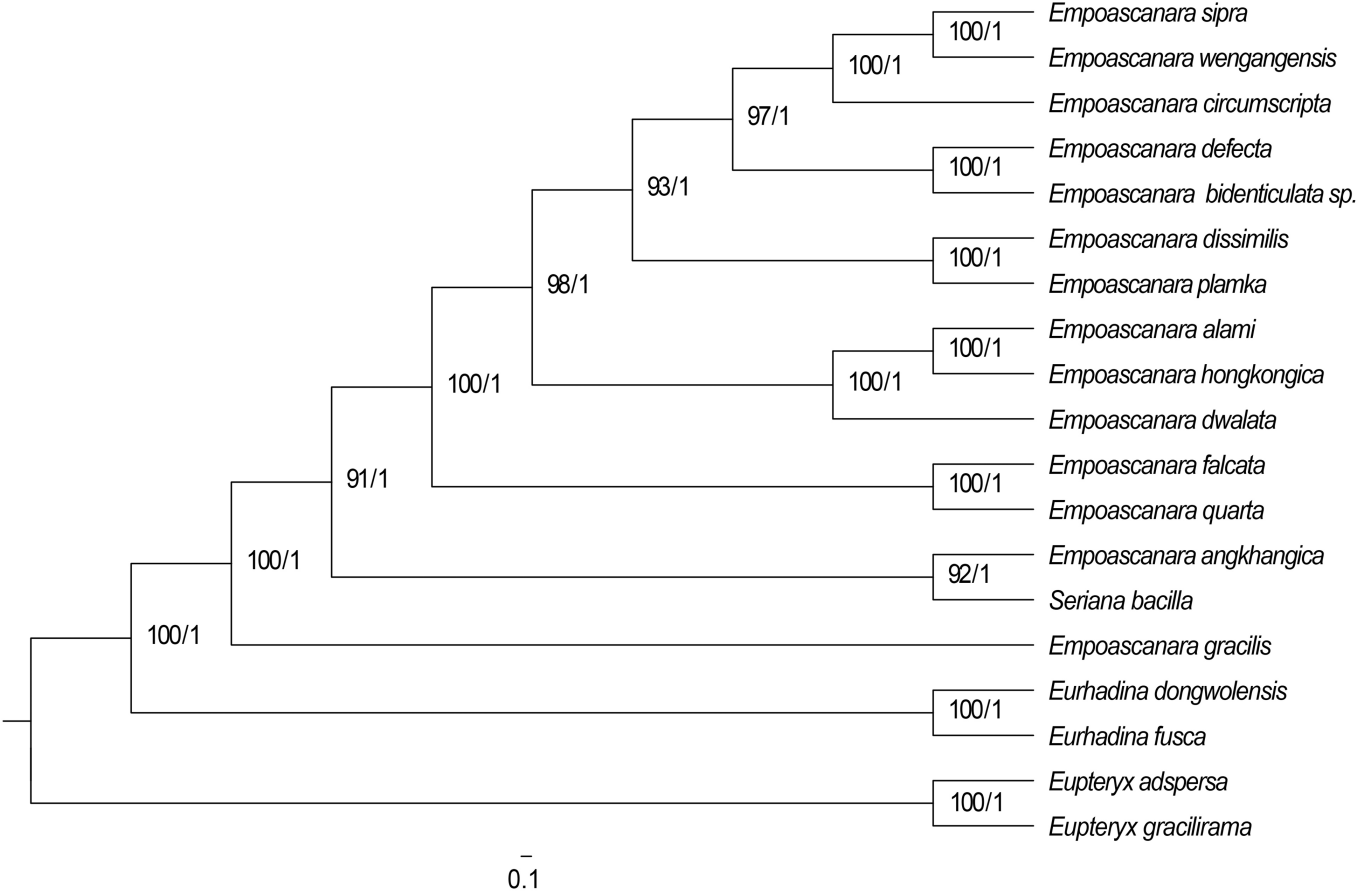
Phylogenetic tree of *S. bacilla* and closely related species constructed based on four mitochondrial genes (COI, COII, Cytb, 16S rRNA). The first and second numbers to the left of each node denote the ultrafast bootstrap value (UBP) for ML analysis and the Bayesian posterior probability (BPP) for Bayesian inference.

In agreement with previous studies, our phylogeny placed *Seriana* and *Empoascanara* as sister groups. To further explore their relationships, we downloaded all available mtDNA sequences for species of these genera from Genbank, and used four species from Typhlocybini as outgroups for a total of 19 species to construct ML and BI phylogenetic trees based on four genes (COI, COII, Cytb, 16S rRNA). The topologies from ML and BI analyses are consistent as follows: *E. gracilis* is sister to the remaining species; *E. sipra*, *E. wengangensis*, and *E. circumscripta* are clustered together; *E. defecta* an*d E. bidenticulatas* sp. n. are sister groups, *E. dissimilis* and *E. plamka* are sister groups; *E. alami*, *E. hongkongica*, and *E. dwalata* are in one clade; *E. falcata* and *E. quarta* are sister groups, and *E. angkhangica* and *S. bacilla* are in one clade, indicating that *Seriana* is closely related to *Empoascanara* (Figure 3).

**FIGURE 4:**
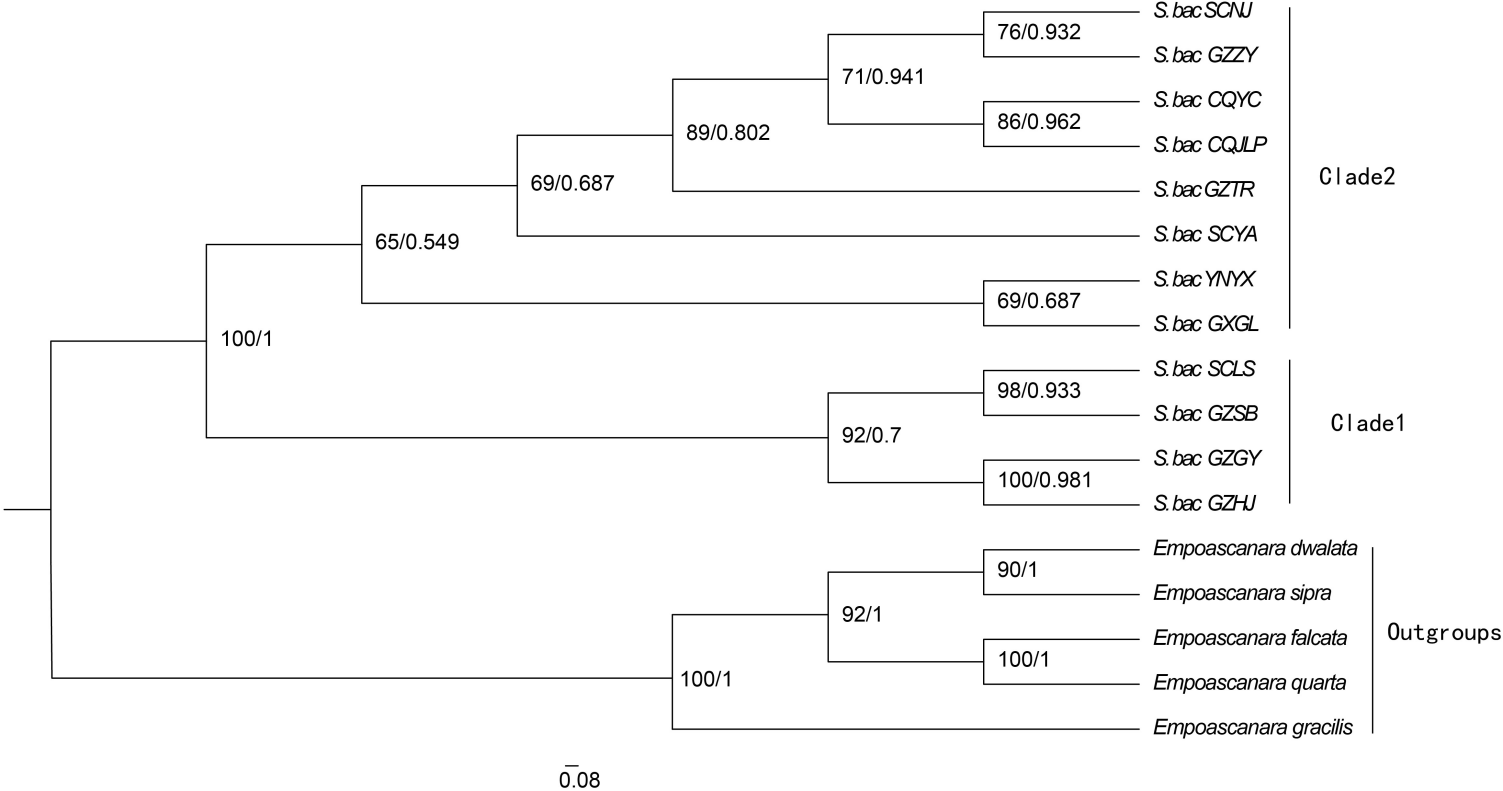
Phylogenetic tree of 12 geographical populations of *S. bacilla* constructed based on combined gene sequences (COI, COII, Cytb, 16S rRNA). The first and second numbers to the left of each node denote the ultrafast bootstrap value (UBP) for ML analysis and the Bayesian posterior probability (BPP) for Bayesian inference.

Five species of *E. dwalata*, *E. sipra*, *E. falcata*, *E. quarta*, and *E. gracilis* were selected as outgroups for ML and BI analyses of the 12 geographical populations of *S. bacilla* based on COI, COII, Cytb and 16S rRNA. According to the clustering of *S. bacilla* in the phylogenetic tree (Figure 4), it was divided into two major branches. Clade 2 was dominated by S. bac CQYC, S. bac CQJLP, S. bac SCNJ, S. bac GZZY, S. bac GZTR, and S. bac SCYA, S. bac YNYX and S. bac GXGL, but support for this branch was low. S. bac SCLS, S. bac GZSB, S. bac GZGY, and S. bac GZHJ were clustered as Clade 1 with 100% support. Except for the two samples from Chongqing, samples from the same region were scattered across multiple clades.

### 3.3 Genetic diversity analysis

The results of analysis based on four genes (COI, COII, Cytb, and 16S rRNA) are shown in Table 5. All populations had high haplotype diversity (1.000), except for Chongqing, where it was low; nucleotide diversity was highest in Guizhou (0.014, 0.024, 0018, 0.008, 0.15), followed by Sichuan (0.01, 0.016, 0.009, 0.008, 0.01), and lowest in Chongqing.

**TABLE 5:** Genetic diversity analysis of different geographical populations of *S. bacilla* calculated based on mitochondrial genes.

Gene	Collection region	Population number	Haplotype diversity	Nucleotide diversity	Average number of nucleotide differences	Number of polymorphic (segregating) sites
COI	GZ	5	1.000	0.014	22.200	41.000
	SC	3	1.000	0.010	15.333	23.000
	CQ	2	0.000	0.000	0.000	0.000
COII	GZ	5	1.000	0.024	16.500	29.000
	SC	3	1.000	0.016	10.667	16.000
	CQ	2	1.000	0.001	1.000	1.000
Cytb	GZ	5	1.000	0.018	20.000	38.000
	SC	3	1.000	0.009	10.667	16.000
	CQ	2	0.000	0.000	0.000	0.000
16S rRNA	GZ	5	1.000	0.008	8.200	17.000
	SC	3	1.000	0.008	8.000	12.000
	CQ	2	0.000	0.000	0.000	0.000
Combined Genes	GZ	5	1.000	0.015	67.000	125.000
	SC	3	1.000	0.010	44.667	67.000
	CQ	2	1.000	0.000	1.000	1.000

Abbreviations: CQ, Chongqing City; GZ, Guizhou Province; SC, Sichuan Province.

The genetic differentiation coefficient (*F*_st_) and gene flow (*N*_m_) of *S. bacilla* were calculated based on different genes using Arlequin software, and the results are shown in Table 6. In the results of COI, 16S rRNA genes and the combined gene in Guizhou and Sichuan, *F*_st_ < 0 (−0.04259, −0.01250 and −0.00872), respectively, and *N*_m_ > 4 (12.24, 40.50 and 57.81), respectively, indicating that there is frequent gene flow and no genetic differentiation between the two populations; in the results of COII and Cytb genes, 0 < *F*_st_ < 0.05, 0.01092, and 0.01489, respectively, and *N*_m_ > 4, 45.28 and 33.07, respectively. Gene flow is frequent, but there is weak genetic differentiation; the results of different gene calculations between Guizhou and Chongqing populations are similar, *F*_st_ > 0.25, 1 < *N*_m_ < 4, indicating that there is some gene flow between the two populations but genetic differentiation is obvious. For Sichuan and Chongqing populations, the *F*_st_ values of the remaining genes are between 0.05 and 0.15, 1 < *N*_m_ < 4, except for the Cytb gene result which is 0.2, 0.15, and 0.25, indicating that there is a moderate genetic differentiation between the two populations.

**TABLE 6:** Genetic differentiation coefficient (*F*_st_) and gene flow (*N*_m_) between collection regions calculated based on different genes.

Gene	Region	GZ	SC	CQ
COI	GZ	–	12.24	1.01
	SC	−0.04259	–	2.88
	CQ	0.33133	0.14815	–
COII	GZ	–	45.28	1.17
	SC	0.01092	–	8.75
	CQ	0.3	0.05405	–
Cytb	GZ	–	33.07	1.03
	SC	0.01489	–	2
	CQ	0.32667	0.2	–
16S	GZ	–	40.50	0.76
	SC	−0.01250	–	3
	CQ	0.39706	0.14286	–
Combined Gene	GZ	–	57.81	1.01
	SC	−0.00872	–	3.11
	CQ	0.33104	0.13836	–

*Note*: Below the diagonal: *F*_st_ values, above the diagonal: *N*_m_ values.

Abbreviations: CQ, Chongqing City; GZ, Guizhou Province; SC, Sichuan Province.

### 3.4 Divergence‐time estimation

**FIGURE 5:**
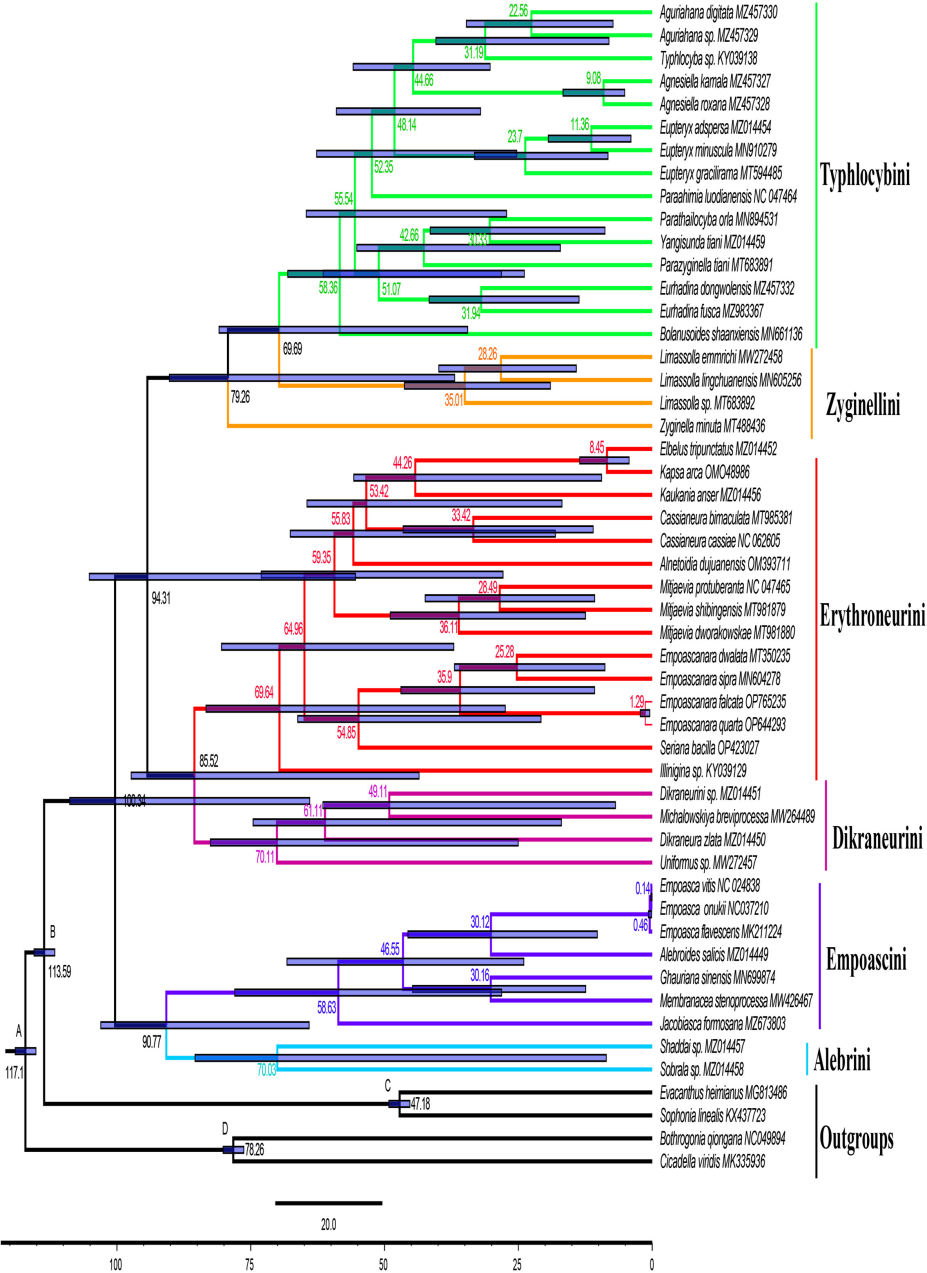
Divergence time tree of Typhlocybinae constructed based on 13 PCGs. (A) root age, <174.1 Mya; (B) 17.5–120 Mya; (C) 17.5–110 Mya; (D) 17.5–90 Mya.

The 13PCGs dataset comprising 47 species of Typhlocybinae was used to estimate divergence times. As shown in the cladogram (Figure 5), the divergence time of Typhlocybinae is 113.59 million years in the middle Cretaceous, and the 95% confidence interval (95% high posterior density, i.e., 95% HPD) is 111.61–115.48 million years; the internal clades begin to diversify at 100.34 million years (95% HPD: 63.98–108.82 million years), the earliest being the Empoascini and Alebrini at 90.77 million years (95% HPD: 64.09–103.01 million years); the Typhlocybini and Zyginellini at 79.26 million years (95% HPD: 36.9–90.21 million years); the Erythroneurini and Dikraneurini are 85.521 million years (95% HPD: 43.54–97.34 million years); and the divergence time of species between the genera of *Seriana* and *Empoascanara* is about 54.85 million years (95% HPD: 20.76–66.23 million years).

**FIGURE 6:**
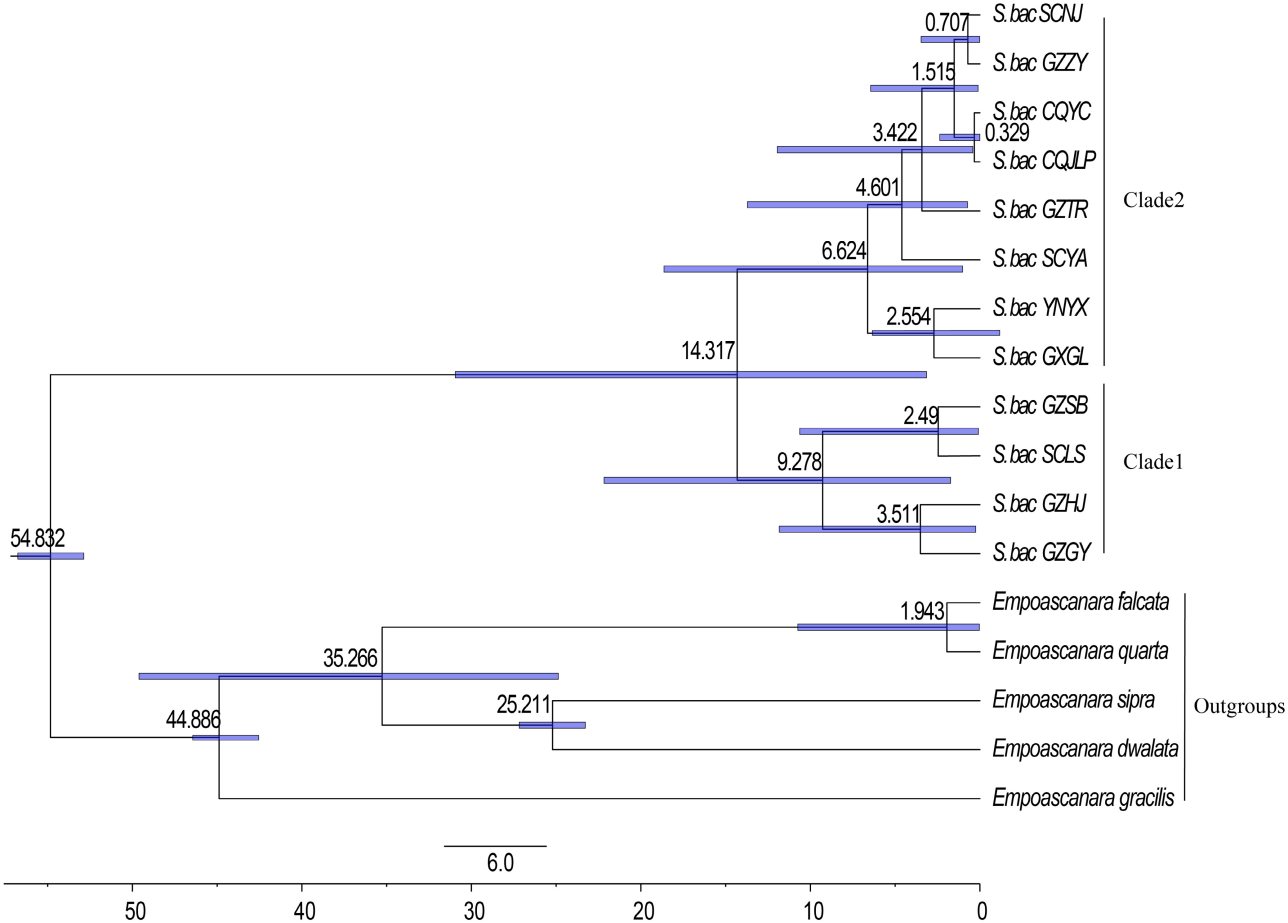
Divergence time tree (Mya) of different geographical populations of *S. bacilla* constructed based on COI, COII, Cytb, and 16S rRNA.

Divergence times were estimated for the different geographic populations of *S. bacilla*, and the main divergence time is shown in Figure 6. The divergence time for *S. bacilla* was about 54.832 Mya (95% HPD: 52.866–56.762 million years) during the Paleoproterozoic period, and this result differs somewhat from the results in Figure 5, but is within the 95% confidence interval. The branches began to evolve about 14.317 Mya (95% HPD: 3.144–30.95 million years) in the Neoproterozoic, with evolutionary clade 1 diverging first and then internally into two branches at about 9.278 million years (95% HPD: 1.723–22.174 million years); evolutionary clade 2 began to differentiate internally in 6.624(95% HPD: 1.021–18.636 million years).

### 3.5 Ancestral area reconstruction

**FIGURE 7:**
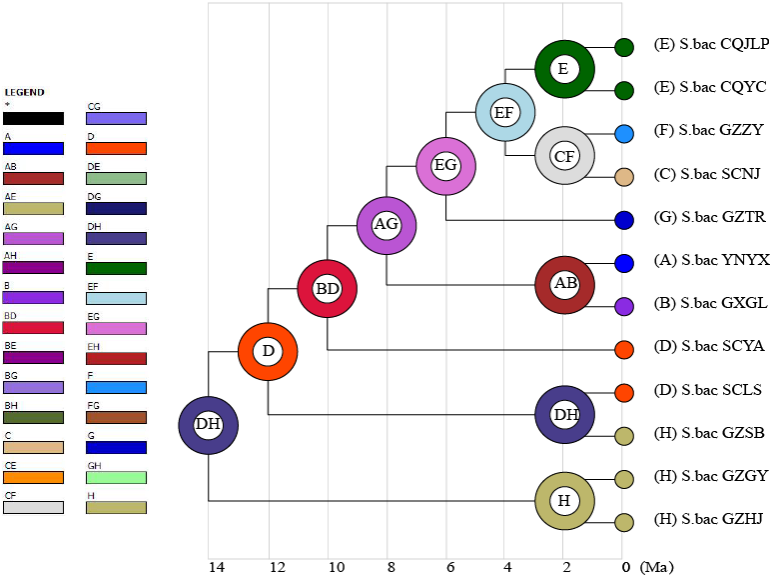
Results of Ancestral area reconstruction of *S. bacilla* based on RASP.

The results of ancestral area reconstruction based on Biogeobears analysis in RASP are shown in Figure 7. Testing and comparison of six models using the AICc weighting method showed that the DIVALIKE + J model was the best‐fitting model. The results indicate that the most recent common ancestor of the sampled populations of *S. bacilla* was most likely distributed in the central region of Guizhou. From there, this species appears to have spread to central Sichuan, then into northeast Guangxi and central Yunnan, to northeast Guizhou. The species then spread to western Chongqing, and finally to northern Guizhou.

### 3.6 Species distribution model

**FIGURE 8:**
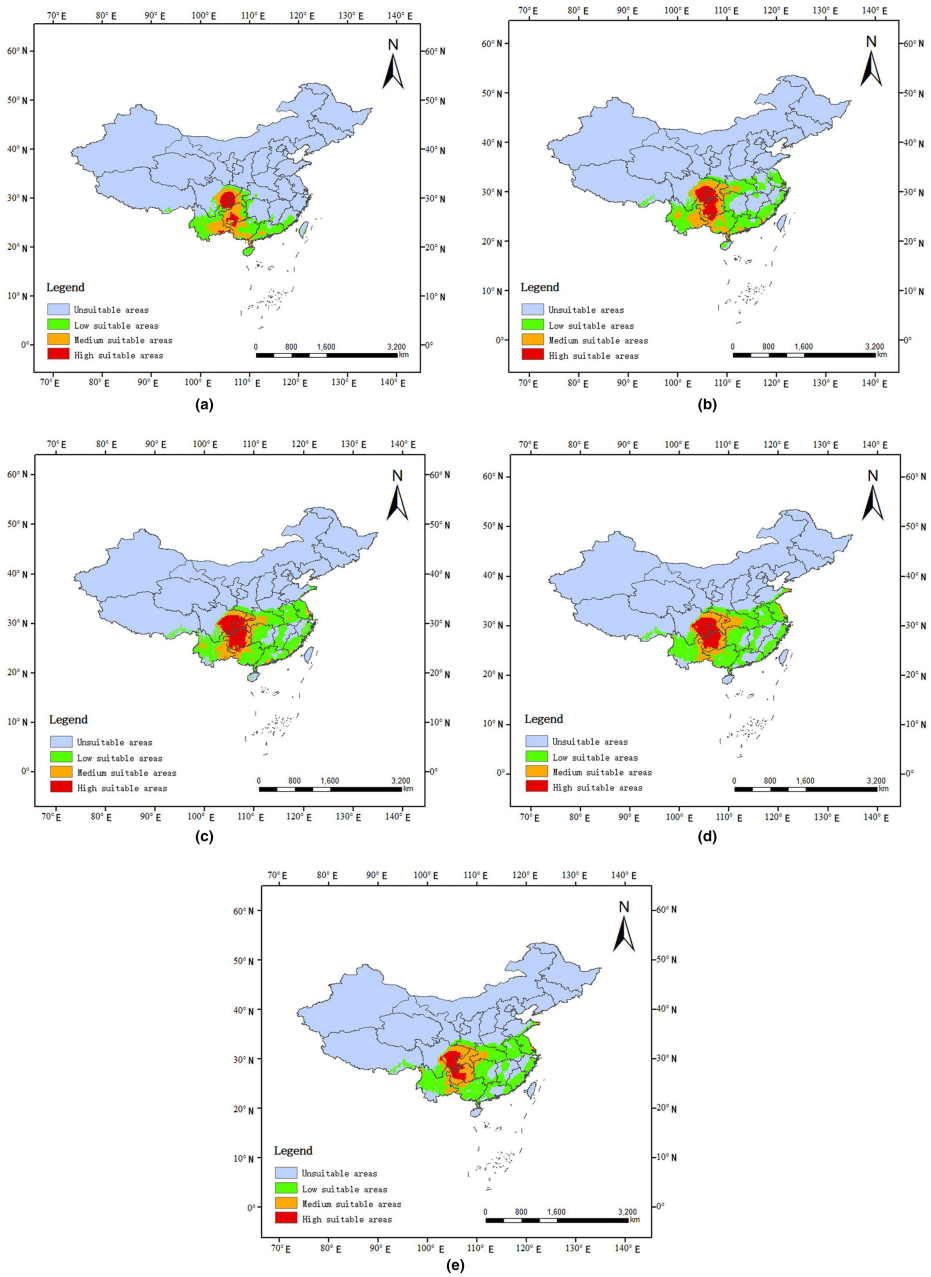
Potential distribution areas of *S. bacilla* in different geological periods using MaxEnt model with bioclimatic factors. (a) Last glacial maximum (LGM); (b), Mid‐Holocene; (c), Present day; (d), year 2050; (e), year 2070.

Figure 8 shows the predicted distributions of *S. bacilla* during different periods. The AUC of the species distribution model is >0.9, indicating that the model has good performance. In LGM, the potential suitable area of *S. bacilla* is 116.250 × 10^4^ km^2^, accounting for 12.109% of the total terrestrial area of China, of which 64.737 × 10^4^ km^2^ is low suitable area, 38.787 × 10^4^ km^2^ is medium suitable area, and 12.726 × 10^4^ km^2^ is high suitable area, with each representing 6.743, 4.040, and 1.326% of Chinese territory, respectively (Table 7). The high suitable areas are mainly concentrated in southeastern Sichuan, southwestern Chongqing, and southwestern Guizhou, with less distribution in Guangxi and Yunnan. By the middle of the Holocene, the potential distribution area of *S. bacilla* increased significantly to 167.624 × 10^4^ km^2^, occupying 17.461% of the total terrestrial area of China, with a total increase of 5.351%. The high suitable area increased by 0.754%, and the middle and low suitable areas both increased by 1.019%. The increased high suitable areas are mainly in southeastern Sichuan and northern Guizhou, the medium suitable areas are mainly in northwestern Yunnan and southwestern Hubei, and the low suitable areas are mainly in Fujian, southeastern Zhejiang and Jiangsu, with some also in Jiangxi, Henan, and Anhui.

**TABLE 7:** The suitable habitat area of *S. bacilla* at different periods predicted based on MaxEnt model.

Climate scenario	Unsuitable area (km^2^)	Low suitable area (km^2^)	Medium suitable area (km^2^)	High suitable area (km^2^)	Proportion of suitable areas (%)
LGM	843.750 × 10^4^	64.737 × 10^4^	38.787 × 10^4^	12.726 × 10^4^	12.109
MH	792.376 × 10^4^	99.089 × 10^4^	48.574 × 10^4^	19.962 × 10^4^	17.461
Current	768.895 × 10^4^	122.955 × 10^4^	39.773 × 10^4^	28.376 × 10^4^	19.907
2050	766.259 × 10^4^	128.283 × 10^4^	40.922 × 10^4^	24.536 × 10^4^	20.181
2070	766.758 × 10^4^	132.691 × 10^4^	44.471 × 10^4^	16.080 × 10^4^	20.129

Abbreviations: LGM, Last glacial maximum in the past; MH, Mid‐holocene.

Compared with the past, the area of both high suitable and low suitable areas of *S. bacilla* in China is larger at present, with 28.376 × 10^4^ km^2^ and 122.955 × 10^4^ km^2^, accounting for 2.956% and 12.808% of the total area, respectively. The low suitable areas increased obviously, while the medium suitable areas showed a decreasing trend, with an area of 39.773 × 10^4^ km^2^ and a proportion of 4.143%. Compared with the Mid‐Holocene, the area of medium suitable areas decreased by 8.801 × 10^4^ km^2^ and the proportion decreased by 0.917%. However, the total area of the suitable areas showed an increasing trend, reaching 191.105 × 10^4^ km^2^, accounting for 19.907% of the total land area of China. In the future, suitable areas for *S. bacilla* in China are predicted to increase. It is worth noting that the suitable habitat for *S. bacilla* is mostly in slightly humid areas. The subtropical monsoon climate with high temperature and rainfall in summer and mild and low rainfall in winter provides good climatic conditions for the survival of *S. bacilla*. The vegetation in the suitable areas is mostly evergreen broad‐leaved forests, and the other rich vegetation types such as evergreen deciduous broad‐leaved mixed forests and shrubs are also important factors affecting the range of their suitable habitat.

## 4 DISCUSSION

In this study, we newly obtained mitochondrial genomes for 10 erythroneurine species and sequenced four genes for representatives of 12 geographical populations of *S. bacilla*. The phylogenetic tree of Typhlocybinae includes all species of all genera of the six tribes recognized in Typhlocybinae for which mitochondrial DNA sequences are available on GenBank. The monophyly of Typhlocybini, Dikraneurini, Empoascini, Erythroneurini, and Alebrini is supported in our results. Song and Li (2014) reported that *Seriana* and *Empoascanara* are very similar in external appearance, with the fuscous body color, the anterior margin of crown produced medially, and usually with an irregular dark spot at anterior margin of the crown medially. Our analysis suggests that *Seriana* is derived from within *Empoascanara* but this result needs to be confirmed by analyses that include more than one species of *Seriana*.

Haplotype diversity was high in all populations of *S. bacilla* except the Chongqing population, with Guizhou having the highest nucleotide diversity, Sichuan the second highest, and Chongqing the lowest. The low genetic diversity from Chongqing may be due to a young population that has not accumulated much genetic variation, or the population may have experienced a “selective sweep” or ‘founder effect’. Generally, older populations have higher genetic diversity and colonizing or invasive populations have lower levels of genetic diversity (Lanzavecchia et al., 2008; Savolainen et al., 2002), and the highest genetic diversity in Guizhou suggests that this population may be the ancestral population of *S. bacilla* in southwest of China, although this may also be an artifact of the larger number of samples from this region.

In the phylogenetic analysis, the Guizhou population is relatively divergent from the Yunnan and Guangxi populations, while the divergence between Yunnan and Guangxi populations is relatively low. The Yunnan‐Guizhou Plateau is a natural geographical barrier between the three populations. The terrain of the Yunnan‐Guizhou Plateau, which is high in the northwest and low in the southeast, may hinder gene flow between Yunnan and Guizhou compared to that between Yunnan and Guangxi. The genetic differentiation coefficient between the two populations in Sichuan and Guizhou is very small (*F*_st_ < 0), and the gene flow is very large (*N*_m_ > 4), indicating that there is no genetic differentiation due to the frequent gene exchange between the two populations. Geographical barriers between Sichuan and Guizhou are small. Based on our results, geographical barriers (or lack thereof) appear to be the main mediators of gene flow among populations of *S. bacilli* in China (Pyron & Burbrink, 2010; Smith et al., 2014; Ye et al., 2013).

On our molecular time tree of Typhlocybinae, the divergence time estimate for Erythroneurini (85.521 million years) is earlier than that of Yan et al. (2022) and later than that of Christopher et al. (2017). The split time between Erythroneurini and Dikraneurini in the study of Yan et al. (2022) is estimated to be 76 million years, while Christopher et al. (2017) reported 95 million years. One possible explanation for these differences is the larger numbers of taxa included in our analysis compared to the previous analyses.

The first fossil Typhlocybinae inclusions from Eocene Rovno amber, and recent molecular time trees place the origin of Cicadellidae in the Cretaceous (Christopher et al., 2023; Johnson, Dietrich, Friedrich, Beutel, et al., 2018; Johnson, Dietrich, Friedrich, Beutelet, et al., 2018; Lu et al., 2021), but most modern genera arising during the Paleogene and multiple transcontinental dispersal events occur in the Paleogene (Cao et al., 2023). The divergence of *S. bacilla* in the southwest was also shown to have occurred about 54.832 Mya during the Paleogene. The divergence of individual populations of this species occurred between 3 and 14 Mya. The divergence within individual branches mostly occurred in the range of 0.329–4.601 million years, with most occurring during the Pleistocene period and a few during the Pliocene. This means that the divergence of the species was mainly influenced by the Quaternary ice age. The climatic upheavals caused by the repeated alternation of Quaternary ice ages had a great impact on the evolution as well as the distribution of flora and fauna in China (Zhang et al., 2016). The uplift of the Tibetan Plateau caused by the collisional compression of the Indian and Eurasian continental plates has greatly altered the environment and climate of southwest China (Favre et al., 2015). During the Pliocene 3–4 million years, the rapid uplift of the Hengduan Mountain Range, the East Asian monsoon was once again strengthened, temperatures dropped, and species migrated to warmer regions. In the Eocene, about 30 Mya, the Sichuan Basin formed due to the uplift of the Qinghai‐Tibet Plateau was separated by Qinling Mountain, Daba Mountain, Yunnan‐Guizhou Plateau and other mountains, and was less affected by glaciers. The climate was mild, providing a favorable living environment for animals and plants during the glacial period (Chen et al., 2011). The Yunnan‐Guizhou Plateau was not covered by glaciers during the Quaternary glacial period, and the impact of the glacial period on the region was relatively small, mainly in terms of temperature and precipitation, while a small range of environmental changes made the species migrate (Hewitt, 2000). It is speculated that the Yunnan‐Guizhou Plateau and the Sichuan Basin are the refuge during the ice age. Around 0.07–0.1 million years, China was in the interglacial period when temperatures began to rise and species migrated from refugia to various locations (Xu et al., 2012).

### 4.1 Taxonomy

#### 4.1.1 *Empoascanara* distant, 1918

*Empoascanara* Distant, 1918: 94.

**Type species.** *Empoascanara prima* Distant, 1918.

Crown is slightly wider, equal in width, or slightly narrower than the widest part of pronotum. Vertex is blunted and slightly produced medially, about half as long as pronotum, often with variable black spots in the central part. Pronotum is often brownish yellow, with orange red tint, or dark only along posterior margin.

**Male genitalia:** Pygofer dorsal appendages with diverse shapes, articulately movable or immovably fused to dorsal margin. Pygofer lobe is smooth or angulated. Subgenital plate expanded basally and characterized by 2–4 macrosetae along outer margin, and bears distinct rigid setae along upper margin form a continuous row from subbase to apex. Style apex varies with preapical lobe distinct. Aedeagus with or without apical, subapical, or basal processes. Connective nearly Y‐shaped, with distinct central lobe and short arms; stem well developed or bifid.

**Distribution:** Afrotropical, Oriental and Australian regions.

#### 4.1.2 *Empoascanara bidenticulata* Luo & Song, sp. nov.

**FIGURE 9:**
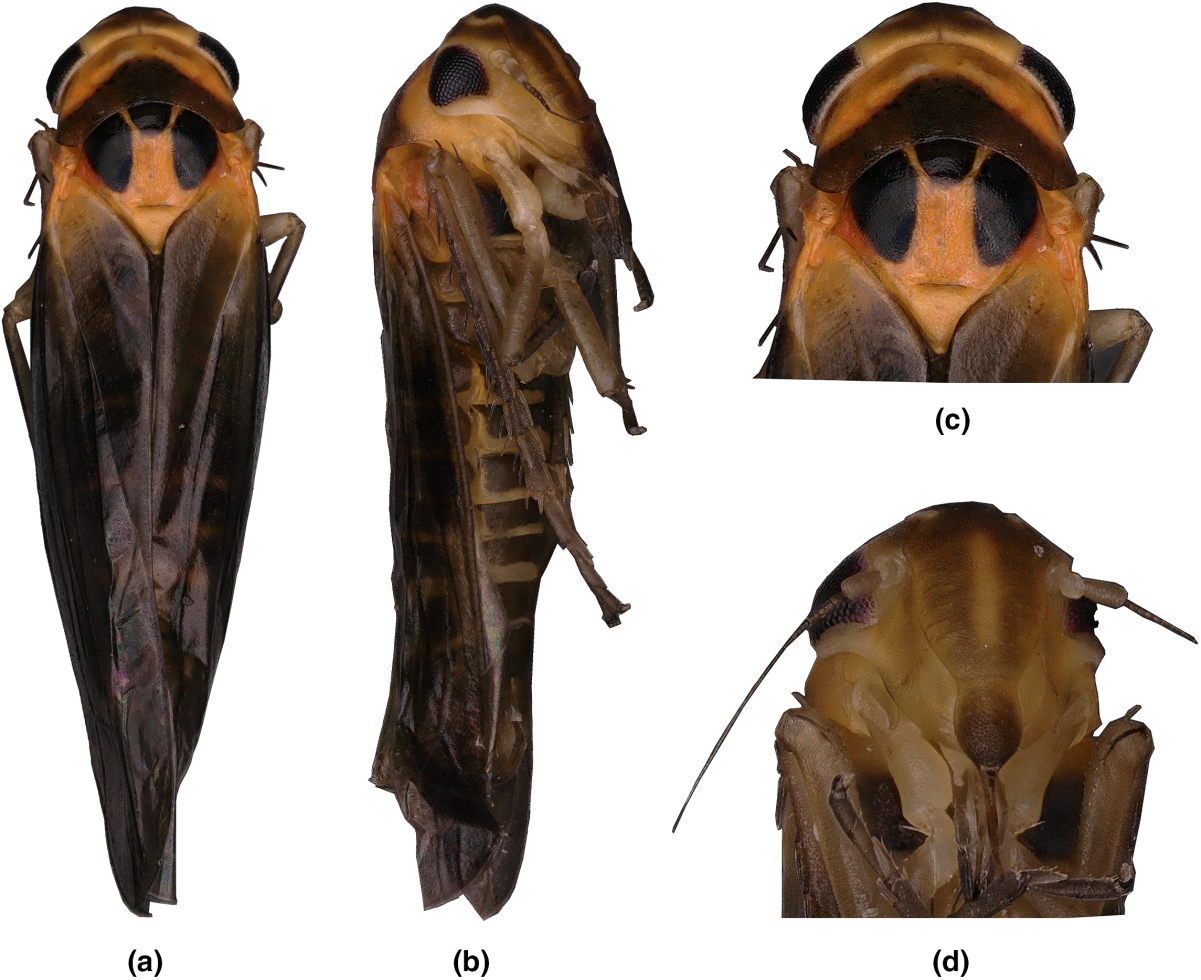
(a–d) *Empoascanara bidenticulata* Luo & Li, sp. nov. (a) habitus, dorsal view; (b) habitus, lateral view; (c) head and thorax, dorsal view; (d) face.

**Description: Description:** Vertex brownish yellow, with a single large black patch in the middle of anterior margin. Eyes black (Figure 9a). Pronotum orange yellow, with dark posterior margin. Scutellum orangeyellow, with basal triangles black. Forewing black brown, with anterior angle near scutellum orange‐yellow (Figure 9).

**FIGURE 10:**
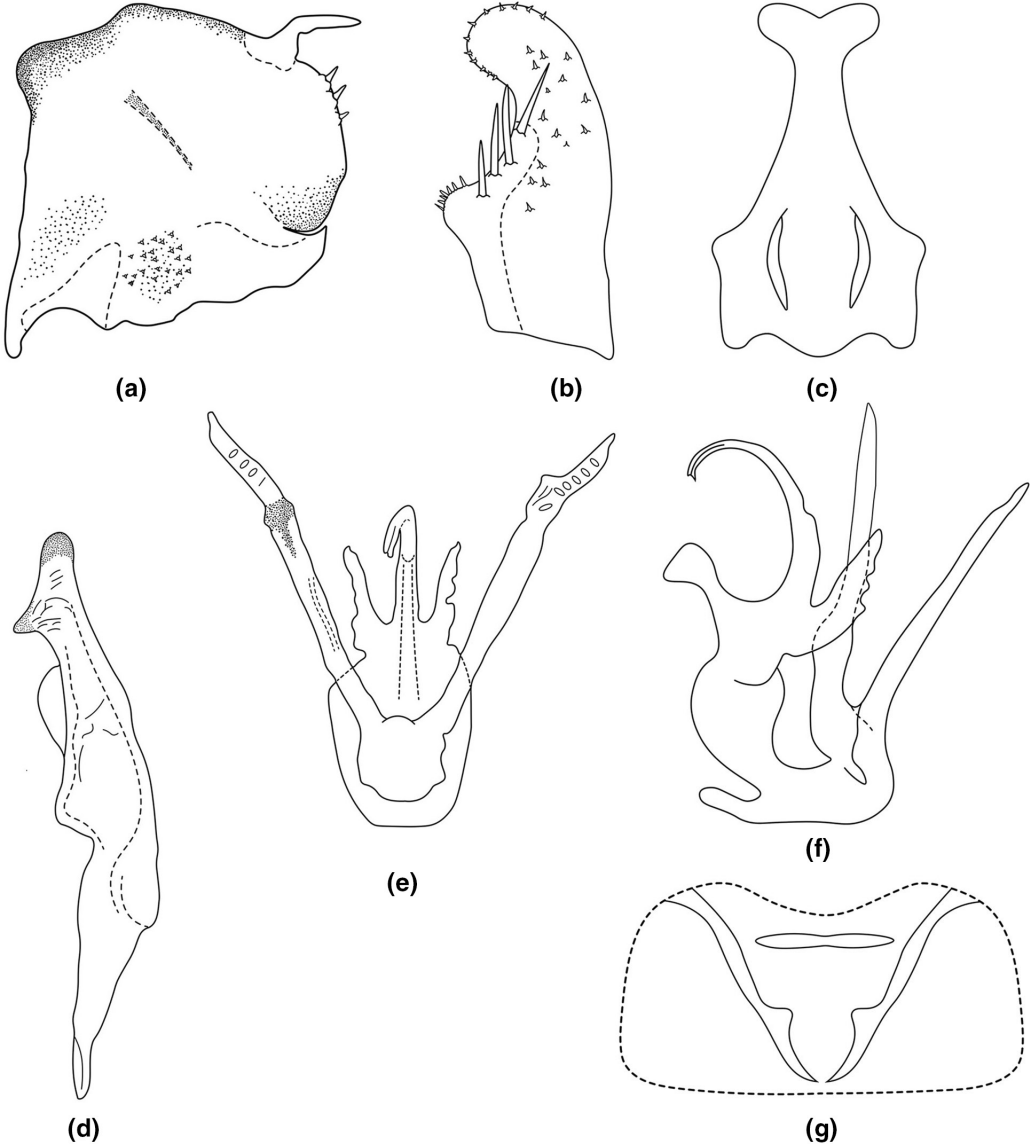
(a–g) *Empoascanara bidenticulata* Luo & Song, sp. nov. (a) Pygofer, lateral view; (b) Subgenital plate; (c) Connective, ventral view; (d) Style, lateral view; (e) Aedeagus, ventral view; (f) Aedeagus, lateral view; (g) Abdominal apodemes, ventral view.

Abdominal apodemes small, not exceeded third sternite (Figure 10g).

**Male genitalia:** Pygofer lobe with well‐developed microtrichia. Pygofer dorsal appendage is unbranched and hook‐like (Figure 10a). Subgenital plate broadened at sub‐base and with four long macrosetae on lateral surface (Figure 10b). Connective Y‐shaped, with stem strong (Figure 10c). Style apex truncate, preapical lobe distinct (Figure 10d). Aedeagus with one pair of large processes basally; aedeagal shaft apex bifurcated; preatrium also with one pair of long processes arising from base. Gonopore located at middle part of the aedeagus shaft, ventrad (Figures 9f, 10e).

**Specimen examined:** Holotype: **♂**, CHINA, Sichuan Prov., Yanyuan County, 5. viii. 2021, coll. Guimei Luo. Paratypes: 42 **♂♂**, 37 **♀♀**, same data as holotype.

**Measurement:** Body length **♂** 2.6–2.8 mm; **♀** 2.5–2.6 mm.

**Remarks:** This species is similar to *E. dissimilis* Dworakowska, 1992, but can be distinguished by the aedeagal shaft bifurcated at apex, with paired long processes arising from base, and its outer margin serrated.

**Etymology:** The specific name is derived from the Latin word “*bidenticulatus*” which refers to the base of aedeagal shaft providing one pair of processes with serrated edges.

## AUTHOR CONTRIBUTIONS

**Guimei Luo:** Conceptualization (equal); data curation (equal); formal analysis (equal); investigation (equal); methodology (equal); resources (equal); software (equal); supervision (equal); validation (equal); visualization (equal); writing – original draft (equal); writing – review and editing (equal). **Tianyi Pu:** Conceptualization (equal); formal analysis (equal); funding acquisition (equal); methodology (equal); software (equal); validation (equal); visualization (equal); writing – review and editing (equal). **Jinqiu Wang:** Conceptualization (equal); data curation (equal); formal analysis (equal); methodology (equal); software (equal); visualization (equal); writing – original draft (equal); writing – review and editing (equal). **Weiwei Ran:** Conceptualization (equal); data curation (equal); formal analysis (equal); resources (equal); software (equal); visualization (equal); writing – original draft (equal). **Yuanqi Zhao:** Conceptualization (equal); data curation (equal); investigation (equal); supervision (equal); validation (equal); writing – review and editing (equal). **Christopher H. Dietrich:** Data curation (equal); formal analysis (equal); investigation (equal); methodology (equal); supervision (equal); validation (equal); writing – review and editing (equal). **Can Li:** Conceptualization (equal); data curation (equal); formal analysis (equal); methodology (equal); resources (equal); supervision (equal); visualization (equal); writing – original draft (equal); writing – review and editing (equal). **Yuehua Song:** Formal analysis (equal); funding acquisition (equal); investigation (equal); methodology (equal); project administration (equal); resources (equal); supervision (equal); validation (equal); visualization (equal); writing – review and editing (equal).

## CONFLICT OF INTEREST STATEMENT

This manuscript has not been published or presented elsewhere in part or in entirety and is not under consideration by another journal. We have read and understood your journal's policies, and we believe that neither the manuscript nor the study violates any of these. There are no conflicts of interest to declare.

## Data Availability

Data supporting the findings of this study are openly available at NCBI (Accession PRJNA987174).
